# MicroRNA-1298-3p inhibits proliferation and invasion of glioma cells by downregulating Nidogen-1

**DOI:** 10.18632/aging.103087

**Published:** 2020-04-30

**Authors:** Xiaohe Xu, Yunchao Ban, Zilong Zhao, Qichen Pan, Jingyu Zou

**Affiliations:** 1 Department of Ophthalmology, Shengjing Hospital of China Medical University, Shenyang 110004, Liaoning, P.R. China; 2 Department of Neurosurgery, The First Affiliated Hospital of China Medical University, Shenyang 110001, Liaoning, P.R. China

**Keywords:** glioma, microRNA-1298-3p, NID1, apoptosis

## Abstract

Glioma is the most prevalent tumor of the central nervous system. To identify differentially expressed miRNAs (DEMs) in gliomas of different grades, bioinformatics analysis was performed. The DEMs between low-grade gliomas (LGGs) and high-grade gliomas (HGGs) were identified by screening the Gene Expression Omnibus and The Cancer Genome Atlas databases using the LIMMA package. Six overlapping DEMs were identified by comparing LGGs and HGGs. Downregulation of miR-1298-3p correlated with poor overall survival rates in glioma patients. Overexpression of miR-1298-3p induced apoptosis of glioma cells and inhibited glioma cell proliferation, migration, and invasion. The basement membrane protein Nidogen-1 (NID1) was identified as a direct binding target of miR-1298-3p in glioma cells. MiR-1298-3p agonist downregulated the NID1 and vimentin levels, but upregulated the level of E-cadherin in glioma cells. Importantly, overexpression of miR-1298-3p induced apoptosis and reduced tumor growth in a mouse xenograft model of glioma. Our results show that miR-1298-3p functions as a tumor suppressor in glioma cells, and suggest that it might serve as a potential biomarker and therapeutic target in glioma patients.

## INTRODUCTION

Glioma is the most prevalent type of cancer of the central nervous system, and accounts for about 80% of malignant brain tumors [1, 2]. Glioma is characterized by a rapid cell proliferation, aggressive invasion, and a poor prognosis [3, 4]. According to the World Health Organization, gliomas are classified into four grades: low-grade gliomas (LGG), grade I pilocytic astrocytoma; grade II diffuse astrocytoma, high-grade gliomas (HGG); grade III anaplastic astrocytoma; and grade IV glioblastoma (GBM) [5, 6]. Among these, GBM is the most aggressive brain tumor with an extremely poor prognosis [7]. Surgery, radiotherapy, and chemotherapy are the main treatment strategies for patients with glioma, but the 5-year survival rates remain about 5% [8–10].

MicroRNAs (miRNAs) are a group of small non-coding RNA molecules that are about 18–25 nucleotides long [11]. MiRNAs regulate gene expression by directly targeting the 3′-untranslated regions (3′-UTRs) of target mRNAs [12]. MiRNAs can silence gene expression by inhibiting translation initiation and inducing degradation of target mRNAs [13]. MiRNAs are involved in different physiological processes, such as cell proliferation, apoptosis, differentiation, metastasis, and tumorigenesis [14]. Recent studies have suggested that miRNAs are involved in glioma tumorigenesis by functioning as oncogenes or tumor suppressors, and might thus serve as diagnostic and prognostic glioma biomarkers [15, 16].

In the present study, we used two bioinformatics analysis tools, the Cancer Genome Atlas (TCGA) and Gene Expression Omnibus (GEO), to identify the miRNAs dysregulated in gliomas, by comparing miRNA expression profiles between low-grade gliomas (LGG) and high-grade gliomas (HGG). Our data show that low levels of miR-1298-3p correlate with poor survival rates in glioma patients. In addition, our results demonstrate that miR-1298-3p inhibits proliferation, migration, and invasion of glioma cells, and suggest that it may serve as a potential biomarker in glioma tumorigenesis.

## RESULTS

### Identification of DEMs in glioma

**Figure 1:**
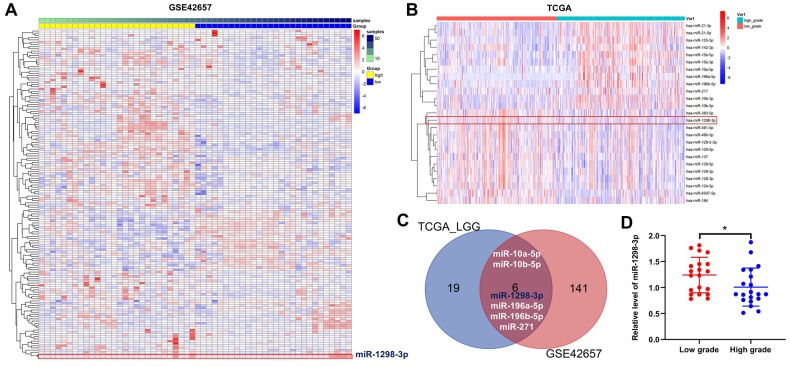
**Identification of DEMs in glioma.** Heat Map showing the miRNA expression profiles in LGG and HGG according to P values, downloaded from (**A**) GSE42657 and (**B**) TCGA datasets. (**C**) Venn diagram of overlapping DEMs from intersection of GSE42657 and TCGA databases. Six overlapping DEMs were identified. (**D**) Relative expression of miR-1298-3p in tumor tissues of patients with LGG and HGG (n = 40). *P < 0.01 compared with the low-grade group.

Raw data from GSE42657 were downloaded from the GEO database and subjected to differential expression analysis using the LIMMA package. In addition, glioma miRNAs expression dataset was downloaded from the TCGA database, and miRNA profile was analyzed using the LIMMA package for LGG and HGG. A total of 147 DEMs were identified in the GSE42657 dataset (Figure 1A), and 25 DEMs were identified in the TCGA dataset (Figure 1B). Using a Venn diagram, 6 overlapping DEMs were identified in the GSE42657 and TCGA datasets (Figure 1C). In addition, RT-qPCR results indicated that obviously decreased expression of miR-1298-3p was observed in the high-grade gliomas group (Figure 1D).

### MiR-1298-3p downregulation correlates with poor survival in glioma

**Figure 2:**
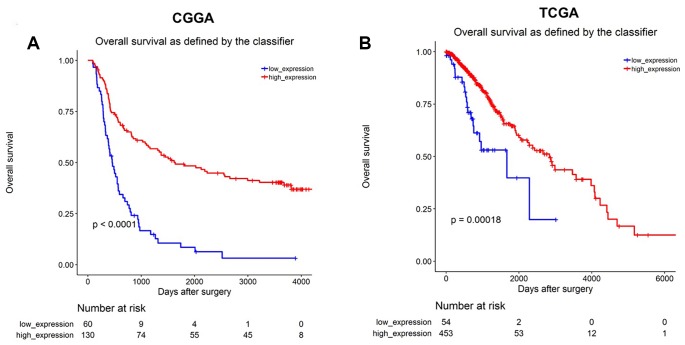
**MiR-1298-3p downregulation correlates with poor survival in glioma.** Association of miR-1298-3p expression with overall survival of glioma patients using (**A**) CGGA and (**B**) TCGA databases. Statistics was calculated using the Kaplan–Meier Plotter online platform.

Next, we analyzed the correlation between the five identified overlapping DEMs and glioma prognosis. Low levels of miR-1298-3p correlated with decreased overall survival rates in glioma patients in the CGGA dataset (Figure 2A). In addition, low levels of miR-1298-3p correlated with poor overall survival rates in glioma patients in the TCGA dataset (Figure 2B). These data indicate that the low miR-1298-3p expression is associated with a poor survival in glioma patients.

### Overexpression of miR-1298-3p inhibits proliferation of glioma cells

**Figure 3:**
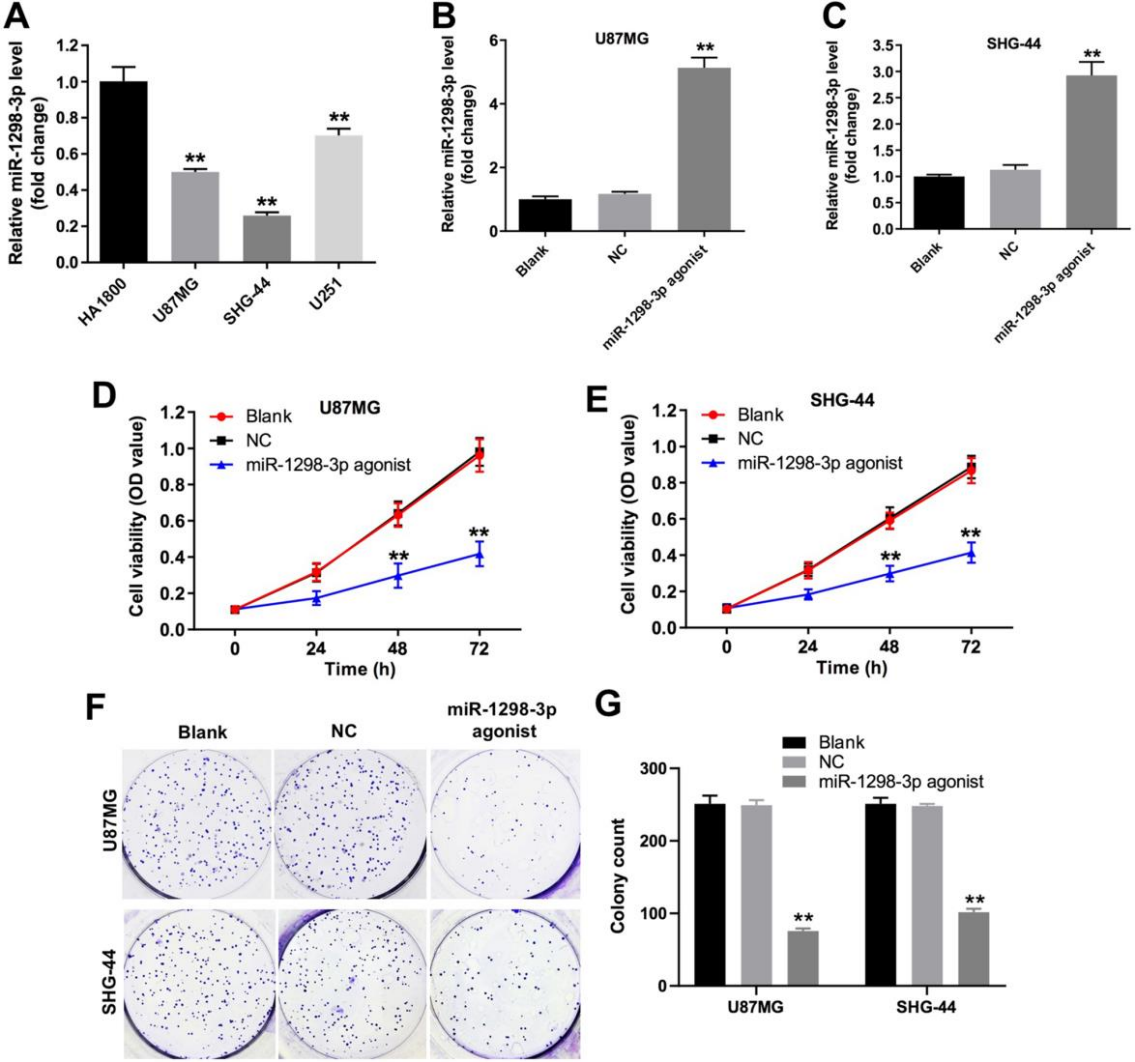
**Overexpression of miR-1298-3p inhibits proliferation of glioma cells.** (**A**) RT-qPCR of miR-1298-3p in cortical astrocytes cell line HA1800, and human glioblastoma cell lines U87MG, SHG-44 and U251. (**B**, **C**) MiR-1298-3p levels analyzed by qRT-PCR in U87MG and SHG-44 cells transfected with miR-1298-3p agonist. (**D**, **E**) Cell viability analyzed by CCK-8 assay in U87MG and SHG-44 cells transfected with miR-1298-3p agonist for 72 h. (**F**, **G**) Cell proliferation analyzed by colony formation assay; **P < 0.01 compared with the NC group.

To investigate the role of miR-1298-3p in glioma cells, we analyzed miR-1298-3p levels in one human cortical astrocytes cell line HA1800, and three glioblastoma cell lines U87MG, SHG-44, and U251, by using RT-qPCR. As shown in Figure 3A, the level of miR-1298-3p was significantly downregulated in U87MG, SHG-44, and U251 cells, compared with HA1800 cells. Since the glioma cell lines U87MG and SHG-44 exhibited the lowest levels of miR-1298-3p, we used them in the next experiments. In addition, the level of miR-1298-3p had no obvious change in human neurons and human microglia groups, compared with HA1800 group (Supplementary Figure 1A). As shown in Figure 3B, 3C, the level of miR-1298-3p was upregulated in U87MG and SHG-44 cells after transfection with miR-1298-3p agonist. Meanwhile, overexpression of miR-1298-3p significantly reduced proliferation of U87MG and SHG-44 cells (Figure 3D–3G). Furthermore, downregulation of miR-1298-3p exhibited a slight increase in the cell proliferation of U251 cells, while overexpression of miR-1298-3p exhibited a significant decrease in the cell proliferation of U251 cells (Supplementary Figure 1B). These data suggested that overexpression of miR-1298-3p inhibits proliferation of glioma cells.

### Overexpression of miR-1298-3p induces apoptosis of glioma cells

**Figure 4:**
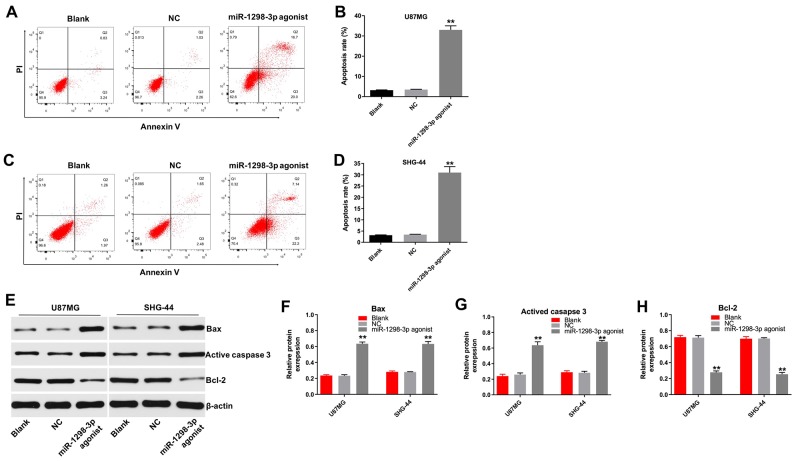
**Overexpression of miR-1298-3p induces apoptosis of glioma cells.** Apoptosis analyzed by Annexin V and PI double staining in (**A**, **B**) U87MG and (**C**, **D**) SHG44 cells transfected with miR-1298-3p agonist for 72 h. (**E**) Western analysis of Bax, active caspase 3, and Bcl-2 levels in U87MG and SHG44 cells. (**F**–**H**) The relative expression of Bax, active caspase 3, and Bcl-2 in U87MG and SHG44 cells, normalized to β-actin; **P < 0.01 compared with the NC group.

The role of miR-1298-3p in the regulation of apoptosis in glioma cells was analyzed by using flow cytometry. As illustrated in Figure 4A–4D, miR-1298-3p agonist markedly induced apoptosis in U87MG and SHG-44 cells. In addition, levels of the pro-apoptotic protein Bax and active caspase 3 were increased, and levels of the anti-apoptotic protein Bcl-2 were decreased in U87MG and SHG-44 cells transfected with miR-1298-3p agonist, compared with NC group (Figure 4E–4H). Moreover, overexpression of miR-1298-3p markedly induced apoptosis in U251 cells as well, while downregulation of miR-1298-3p slightly increased apoptosis of U251 cells (Supplementary Figure 1C, 1D). These data indicate that overexpression of miR-1298-3p induces apoptosis of glioma cells.

### Overexpression of miR-1298-3p inhibits migration and invasion of glioma cells

**Figure 5:**
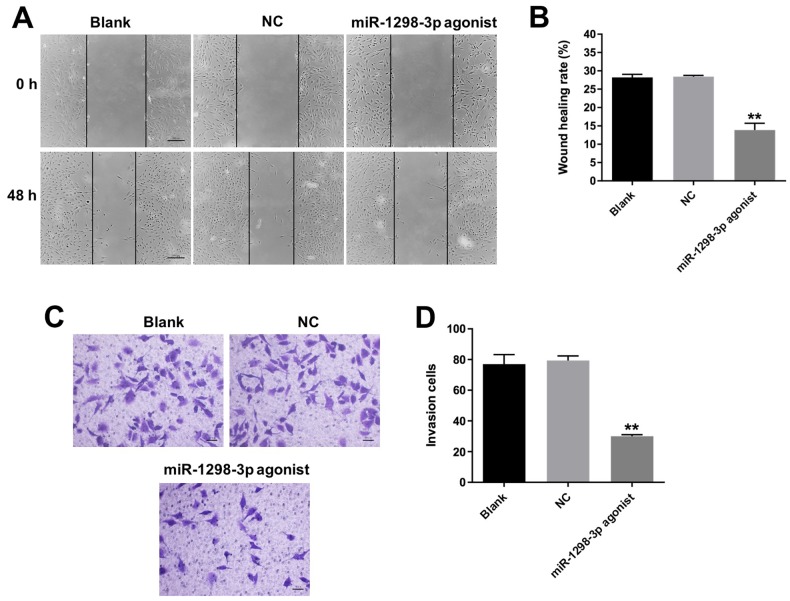
**Overexpression of miR-1298-3p inhibits migration and invasion of glioma cells.** (**A**, **B**) Migration of U87MG cells transfected with miR-1298-3p agonist for 48 h, analyzed by wound healing assay. (**C**, **D**) Invasion ability of U87MG cells transfected with miR-1298-3p agonist for 24 h, analyzed by transwell invasion assay; **P < 0.01 compared with the NC group.

Next, we analyzed the effect of miR-1298-3p on U87MG cell migration using the wound healing assay. As shown in Figure 5A and 5B, upregulation of miR-1298-3p significantly suppressed the migration ability of U87MG cells. In addition, the transwell invasion assay showed that miR-1298-3p agonist markedly inhibited the invasion ability of U87MG cells (Figure 5C, 5D). Furthermore, overexpression of miR-1298-3p obviously inhibited the migration and invasion abilities of U251 cells as well, while downregulation of miR-1298-3p slightly promoted the migration and invasion abilities of U251 cells (Supplementary Figure 1E–1H). These results indicate that overexpression of miR-1298-3p inhibits migration and invasion of glioma cells.

### Nidogen-1 is a direct binding target of miR-1298-3p

**Figure 6:**
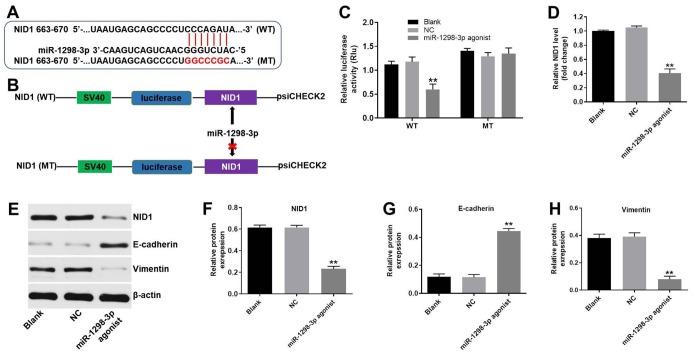
**NID1 is a direct binding target of miR-1298-3p.** (**A**, **B**) Sequence alignment of miR-1298-3p with the binding sites within the WT or MT regions of NID1. (**C**) Luciferase activity in U87MG cells following co-transfection with NID1-WT/MT 3’-UTR plasmid and miR-1298-3p agonist measured using dual luciferase reporter assay. (**D**) RT-qPCR of NID1 levels in U87MG cells transfected with miR-1298-3p agonist. (**E**) Western analysis of NID1, E-cadherin, and vimentin protein levels in U87MG cells. (**F**–**H**) The relative expression of NID1, E-cadherin, and vimentin in U87MG cells normalized to β-actin; **P < 0.01 compared with the NC group.

To identify the target genes of miR-1298-3p, we used the online bioinformatics tools TargetScan (http://www.targetscan.org/vert_71/) and miRDB (http://www.mirdb.org). As shown in Figure 6A, Nidogen-1 (NID1) was identified as a potential target of miR-1298-3p. To validate that NID1 is indeed a target of miR-1298-3p, we used a dual-luciferase reporter assay. As shown in Figure 6B, 6C, miR-1298-3p agonist suppressed the luciferase activity of NID1-WT, but it did not affect the luciferase activity of NID1-MT. Overexpression of miR-1298-3p significantly decreased the NID1 gene expression in U87MG cells (Figure 6D). In addition, miR-1298-3p agonist markedly decreased the protein levels of NID1 and vimentin, but increased the protein level of E-cadherin in U87MG cells, compared with NC group (Figure 6E–6H). These data indicate that NID1 is a direct target of miR-1298-3p.

### Upregulation of miR-1298-3p inhibits tumorigenesis of U87MG subcutaneous xenografts *in vivo*

**Figure 7:**
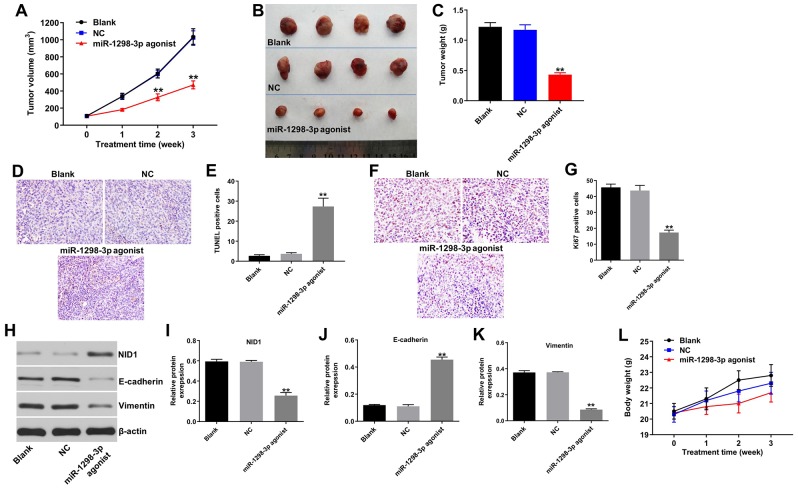
**Upregulation of miR-1298-3p inhibits tumorigenesis of U87MG subcutaneous xenografts *in vivo*.** U87MG cells were subcutaneously injected into nude mice, and 50 nM miR-1298-3p agonist was directly injected into the tumors twice a week. (**A**) Xenograft tumor volume was monitored weekly. (**B**, **C**) Xenografts tumors were photographed and calculated. (**D**, **E**) TUNEL assay of apoptosis in tumor tissues. (**F**, **G**) IHC assay of proliferation in tumor tissues. (**H**) Protein levels of NID1, E-cadherin, and vimentin in tumor tissues analyzed by western blotting. (**I**–**K**) The relative expression of NID1, E-cadherin, and vimentin in tumor tissues quantified, and normalized to β-actin. (**L**) The body weights of mice were monitored. **P < 0.01 compared with the NC group.

To explore the role of miR-1298-3p in regulating the glioma tumor growth *in vivo*, U87MG and U251 subcutaneous xenograft models were established. As shown in Figure 7A–7C and Supplementary Figure 2A–2C, miR-1298-3p agonist treatment significantly inhibited the tumor volume and tumor weight of U87MG or U251 subcutaneous xenografts, compared with NC group. In addition, TUNEL assay showed that overexpression of miR-1298-3p markedly induced apoptosis in tumor tissues, compared with the NC group (Figure 7D, 7E). Moreover, IHC assay indicated that overexpression of miR-1298-3p notably inhibited proliferation in tumor tissues, compared with the NC group (Figure 7F, 7G). Meanwhile, miR-1298-3p agonist markedly decreased the protein levels of NID1 and vimentin, but increased the protein level of E-cadherin in tumor tissues, compared with NC group (Figure 7H–7K). Furthermore, the body weights of mice had no obvious change between miR-1298-3p agonist group and NC group, indicating that miR-1298-3p had no system toxicity on mice (Figure 7L and Supplementary Figure 2D). These data show that upregulation of miR-1298-3p inhibits the glioma tumorigenesis *in vivo*.

## DISCUSSION

Previous studies have indicated that glioma cells that survive the initial therapies contribute to tumor recurrence and glioblastoma pathogenesis [17]. Therefore, identifying the molecular mechanisms and potential glioma biomarkers is urgently needed. MiRNAs play an important role in the development and progression of human cancer [18], and may act as prognostic factors and potential therapeutic targets in gliomas [19]. In the present study, miRNA expression profiling of LGG vs. HGG gliomas was performed by bioinformatics data analysis. Two miRNA expression profile datasets (GSE42657 dataset and TCGA dataset) were used to identify the potential biomarkers in glioma. Six overlapping DEMs between LGG and HGG were identified. Our data indicated that the level of miR-1298 was down-regulated HGG tissues in both GSE42657 and TCGA datasets. In addition, survival analysis using the CGGA and TCGA datasets revealed that the low level of miR-1298 correlated with poor glioma survival rates. Moreover, RT-qPCR results indicated that obviously decreased expression of miR-1298 was observed in the high-grade gliomas group. However, the level of miR-1298 was up-regulated HGG tissues in GSE11209 dataset. GSE11209 dataset includes 10 low-grade gliomas and 15 high-grade gliomas, while the TCGA dataset includes 54 low-grade gliomas and 453 high-grade gliomas. These data indicated that low miR-1298 expression was observed in most HGG tissues.

Previously, a lower miR-1298-3p expression was associated with poor overall survival rates and lymph node metastasis in patients with gastric cancer [20]. Moreover, the level of miR-1298 was downregulated in bladder cancer tissues, implying that miR-1298 might be a diagnostic marker of bladder cancer [21]. Our study demonstrates that overexpression of miR-1298-3p inhibits proliferation, migration, and invasion of glioma cells. Moreover, our data show that upregulation of miR-1298-3p induces glioma cell apoptosis *in vitro* and *in vivo*, and indicate that overexpression of miR-1298-3p inhibits the glioma tumorigenesis.

MiRNAs exert their functions by suppressing expression of their target genes [22]. Our data identify Nidogen-1 (NID1) as a binding target of miR-1298-3p. NID1 is a basement membrane glycoprotein that can form collagen- and laminin-containing networks [23]. Upregulation of NID1 promotes proliferation and metastasis in endometrial and lung cancer [24, 25]. NID1 is mainly generated by mesenchymal cells [26]. A previous study has indicated that NID1 promotes the epithelial-mesenchymal transition (EMT) in ovarian cancer [27]. The process of EMT is complex; cells lose their epithelial polarity and acquire invasive properties of mesenchymal cells, which gain an increased invasive phenotype [28]. Loss of the epithelial marker E-cadherin and gain of the mesenchymal marker vimentin are the critical steps during EMT [27]. We have found that overexpression of miR-1298-3p downregulates the levels of NID1 and vimentin, and upregulates the level of E-cadherin in glioma cells, indicating that overexpression of miR-1298-3p inhibits glioma cell invasion. These data indicate that miR-1298-3p inhibits proliferation and invasion of glioma cells by regulating the NID1 expression, as well as the downstream targets vimentin and E-cadherin.

Together, our data show that the low level of miR-1298-3p correlates with a poor prognosis in glioma patients. In addition, our results indicate that miR-1298-3p functions as a tumor suppressor that inhibits proliferation and invasion of glioma cells by suppressing the NID1 expression, and suggest that miR-1298-3p might serve as a potential biomarker for the glioma treatment. However, in the future, a deeper understanding of the molecular mechanisms regulating tumor growth by miR-1298-3p/NID1 axis require further investigation.

## MATERIALS AND METHODS

### Clinical specimens

Twenty pairs of LGG tissue and HGG tissues samples were collected from glioma patients who underwent surgical resection between 2016 and 2018 at First Affiliated Hospital of China Medical University. The present study was approved by the Ethics Committee of the First Affiliated Hospital of China Medical University. Written informed consents were obtained by participants.

### Data collection

GSE42657 dataset containing miRNAs expression data of LGG and HGG was downloaded from the GEO database (https://www.ncbi.nlm.nih.gov/geo/). Glioma miRNAs expression data from LGG and HGG were downloaded from the TCGA portal (http://tcga-data.nci.nih.gov/tcga).

### Differential expression analysis

The LIMMA package was used to screen differentially expressed miRNAs (DEMs) between LGG and HGG. The overlapping DEMs were identified using a Venn diagram from the GSE42657 and TCGA datasets; P-value <0.05 was considered significant.

### Survival analysis

To assess the prognostic value of the overlapping DEMs, survival analysis was performed, based on the CGGA (http://www.cgga.org.cn) and TCGA (http://ualcan.path.uab.edu/index.html) datasets; P<0.05 was regarded as statistically significant. The optimum cut-off of the overall survival curve was determined using X-tile. X-tile plots provide a single and intuitive method to assess the association between variables and survival. The X-tile program can automatically select the optimum data cut point according to the highest χ² value (minimum p value) defined by Kaplan–Meier survival analysis and log-rank test. X-tile plots were determined using the X-tile software version 3.6.1 (Yale University School of Medicine, New Haven, CT, USA).

### Cell culture and transfections

Human glioblastoma cell lines U87MG, SHG-44, and U251 were purchased from Type Culture Collection of the Chinese Academy of Sciences (Shanghai, China). Human cortical astrocytes cell line HA1800, human neurons and human microglia were purchased from ScienCell Research Laboratories, Inc. (Carlsbad, CA, USA). Cells were cultured in DMEM medium (Thermo Fisher Scientific, MA, USA) supplemented with 10% fetal bovine serum (FBS, Thermo Fisher Scientific) and 100 U/mL penicillin/streptomycin. The third generation of the cells was used in the study.

MiR-1298-3p agonist (5’-CAUCUGGGCAACUGACUGAAC-3’), miR-1298-3p antagonist (5’-GUUCAGUCAGUUGCCCAGAUG-3’) and agonist NC, purchased from GenePharma (Shanghai, China). 10 nM miR-1298-3p agonist, 10 nM miR-1298-3p antagonist, or 10 nM NC were transfected into U87MG and SHG-44 cells using Lipofectamine 2000 (Thermo Fisher Scientific) according to the manufacturer’s protocol. The culture medium containing 10% FBS was changed 6 h after transfection, and cells were then incubated for 42 h at 37°C.

### Reverse transcription-quantitative polymerase chain reaction (RT-qPCR)

TRIzol reagent (Thermo Fisher Scientific) was used to extract total RNA according to the manufacturer's protocol. The PrimeScript RT Reagent Kit (Takara Bio Inc. Shiga, Japan) and EntiLink™ 1^st^ Strand cDNA Synthesis Kit (ELK Biotechnology, Wuhan, China) were used to synthesize complementary DNA (cDNA). RT-qPCR was performed using SYBR Premix Ex Taq II kit (Takara) on StepOnePlus RT-PCR system (Applied Biosystems, CA, USA) using the following conditions: 95°C for 30 sec, 40 cycles at 95°C for 30 sec, and 60°C for 30 sec. The primers used were as follows: miR-1298-3p: F, 5’-CATCTGGGCAACTGACTGAAC-3’; R, 5’-CTCAACTGGTGTCGTGGAGTC-3’. NID1, F, 5’-ATTCCCAGTCAGAGGCGCTAT-3’; R, 5’-GAACTTGTCTTCCCGTCGTGT-3’. U6: F, 5’-CTCGCTTCGGCAGCACAT-3’; R, 5’-AACGCTTCACGAATTTGCGT-3’. Actin: F, 5’-GTCCACCGCAAATGCTTCTA-3’; R, 5’-TGCTGTCACCTTCACCGTTC-3’. Expression of miR-1298-3p was normalized to U6, and expression of NID1 was normalized to actin, using the 2^−ΔΔCt^ method.

### Cell proliferation assay

Cell viability was measured using the Cell Counting Kit (CCK)-8 (Beyotime, Shanghai, China). 5 × 10^3^ U87MG or SHG-44 cells were plated onto 96-well plates and incubated at 37°C overnight. Cells were then transfected with miR-1298-3p agonist for 72 h. Subsequently, 10 μL of CCK-8 reagent was added into each well, cells were incubated for another 2 h, and absorbance at 450 nm was measured using a microplate reader (Bio-Rad).

### Colony formation assay

5 × 10^3^ U87MG or SHG-44 cells were plated into 6-well plates and incubated at 37°C overnight. Cells were then transfected with miR-1298-3p agonist (3 days, 37°C) for 2 weeks, stained with methylene blue (room temperature, 1 h), photographed using a fluorescence microscope (Olympus, Tokyo, Japan), and the number of cell colonies and clusters were counted.

### Cell apoptosis assay

Flow cytometry was performed to determine apoptosis of glioma cells. Cells were washed with cold PBS (Thermo Fisher Scientific), and incubated with 100 μL of RNase (100 mg/L) at 37°C for 30 min. After that, cells were stained with 5 μL of propidium iodide (PI) and 5 μL of Annexin V-FITC for 15 min at room temperature in a darkness. The percentage of apoptotic cells was detected on a flow cytometer (FACScan™, BD Biosciences, Franklin Lakes, NJ, USA).

### Western blotting

Cells were lysed in ice-cold RIPA buffer (Thermo Fisher Scientific), and the total amount of protein was quantified using the BCA method (Beyotime Institute of Biotechnology). Proteins (30 μg per lane) were separated by 10% SDS-PAGE, and transferred onto polyvinylidene difluoride (PVDF) membrane (Thermo Fisher Scientific). The membrane was blocked in 5% skimmed milk for 1 h at room temperature. After washing in TBST, the membrane was incubated (4°C, overnight) with the following primary antibodies: anti-Bax (1:1000, Abcam), anti-active caspase 3 (1:1000, Abcam), anti-Bcl-2 (1:1000, Abcam), anti-NID1 (1:1000, Abcam), anti-E-cadherin (1:1000, Abcam), anti-Vimentin (1:1000, Abcam), and anti-β-actin (1:1000, Abcam). After washing, the membrane was then incubated (room temperature, 1 h) with a secondary antibody (1:5000, Abcam). The signal was visualized using chemiluminescence (Thermo Fisher Scientific); β-actin was used as an internal control.

### Wound healing assay

U87MG cells were seeded at a density of 5×10^5^ cells into a 12-well culture plate overnight at 37 °C. At 80% confluence, a wound area was made in the cell monolayer with a 20 μL pipette tip. Subsequently, the cells were washed with PBS and cultured with fresh FBS free medium at 37°C. Then, cells were transfected with 10 nM miR-1298-3p agonist for 48 h at 37°C. The width of the wound area was photographed at 0 h and 48 h using a fluorescence microscope (Olympus), and cell migration was quantified using ImageJ software.

### Transwell invasion assay

Matrigel (BD Biosciences, NJ, USA) was added into the upper chamber, and overlaid with U87MG cells suspended in 100 μL of serum-free media. 600 μL of DMEM medium supplemented with 10% FBS was added into the lower compartment as a chemoattractant. After 24 h, cells attached to the upper membrane were removed using a cotton swab. Invading cells were fixed with 4% paraformaldehyde, stained with 0.2% crystal violet, and photographed using a laser confocal microscope (Olympus).

### Dual-luciferase reporter assay

Oligonucleotides containing wild-type (WT) or mutant (MT) miR-1298-3p binding sites of 3’-UTR of the NID1 mRNA cDNA fragment were ligated into the psiCHECK-2 vector (Promega, Corp., Singapore). NID1 reporter construct (WT or MT) was co-transfected with miR-1298-3p agonist or agonist NC using Lipofectamine 2000 (Thermo Fisher Scientific). After 48 h, the luciferase activity in cell lysates was detected using Dual-Luciferase Reporter Assay System (Promega Madison, WI) according to the manufacturer’s protocol, using the renilla luciferase activity as an endogenous control.

### Animal study

4-6-weeks old BALB/c nude mice were purchased from the Shanghai SLAC Animal Center (Shanghai, China), and randomized into 3 groups: Blank, NC, and miR-1298-3p agonist group. U87MG cells were resuspended in matrigel, and then 5 × 10^6^ U87MG cells were injected subcutaneously into the left flank of nude mice. When the tumors reached about 100 mm^3^, 50 nM miR-1298-3p agonist was directly injected into the tumors twice a week. The tumor size was calculated using the formula V = (length x width^2^)/2 (Width < Length). After 21 days of treatment, mice were sacrificed, and the entire tumors were weighed**.** All animal experiments were approved by the Institutional Ethical Committee of the First Affiliated Hospital of China Medical University, and animals were maintained following the guidelines of the Institutional Animal Care and Use Committee**.**

### TUNEL staining assay

Deparaffinized tissue sections were stained using an APO-BrdU™ TUNEL Assay Kit (Thermo Fisher Scientific) according to the manufacturer’s instructions.

### Immunohistochemistry (IHC) assay

Deparaffinized tissue sections were incubated with the Ki67 monoclonal antibody overnight at 4°C, and then incubated with biotinylated goat anti-rabbit IgG for 30 min at room temperature. The IHC detection system (EnVision kit; Dako Japan) was used to visualize IHC reactions.

### Statistical analysis

All experiments were repeated three times. Data are presented as the mean ± standard deviation (S.D.). All statistical analyses were performed using GraphPad Prism software (version 7.0, La Jolla, CA, USA). One-way analysis of variance (ANOVA) and Tukey’s tests were carried out for multiple group comparisons. The results were considered significant at *P < 0.05.

## Supplementary Material

Supplementary Figures
