# Supplementary material for: MicroRNA-1298-3p inhibits proliferation and invasion of glioma cells by downregulating Nidogen-1
Source: Aging (Albany NY). 2020 Apr 30;12(9):7761–73. doi: 10.18632/aging.103087 (PMC7244082; doi:10.18632/aging.103087)
Supplement: Supplementary Figures [file aging-12-103087-s001..pdf]

SUPPLEMENTARY FIGURES

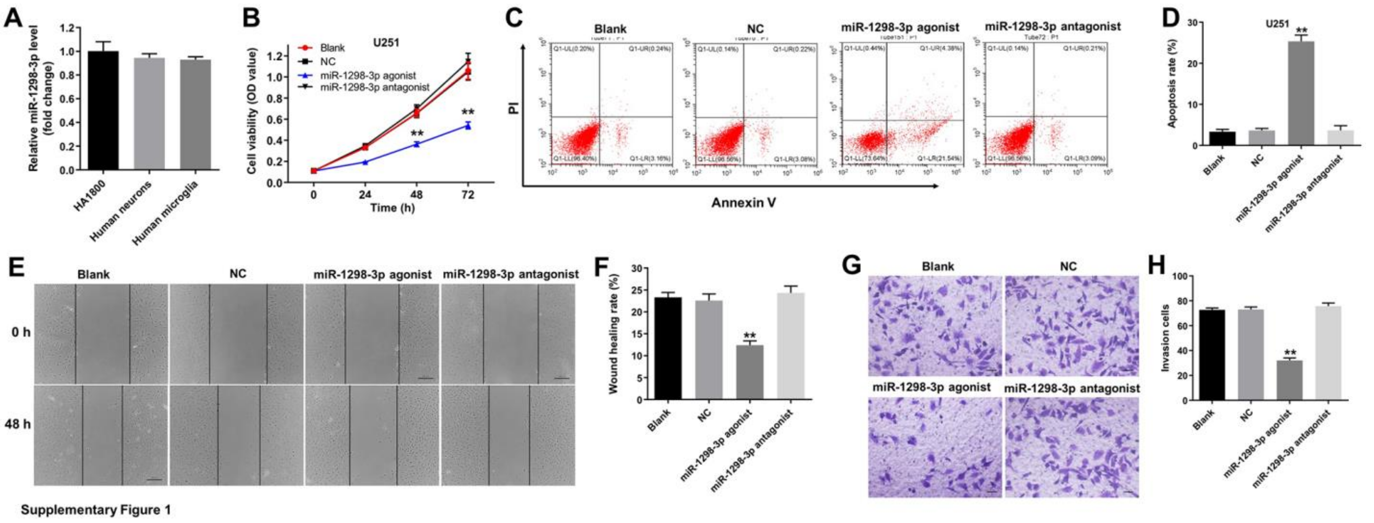

Supplementary Figure 1. Overexpression of miR-1298-3p inhibits the growth of U251 cells. (**A**) RT-qPCR of miR-1298-3p in HA1800, human neurons and human microglia. (**B**) Cell viability analyzed by CCK-8 assay in U251 cells transfected with miR-1298-3p agonist or miR-1298-3p antagonist for 72 h. (**C**, **D**) Apoptosis analyzed by Annexin V and PI double staining in U251 cells transfected with miR-1298-3p agonist or miR-1298-3p antagonist for 72 h. (**E**, **F**) Migration of U251 cells transfected with miR-1298-3p agonist or miR-1298-3p antagonist for 48 h, analyzed by wound healing assay. (**G**, **H**) Invasion ability of U251 cells transfected with miR-1298-3p agonist or miR-1298-3p antagonist for 24 h, analyzed by transwell invasion assay; **P < 0.01 compared with the NC group.

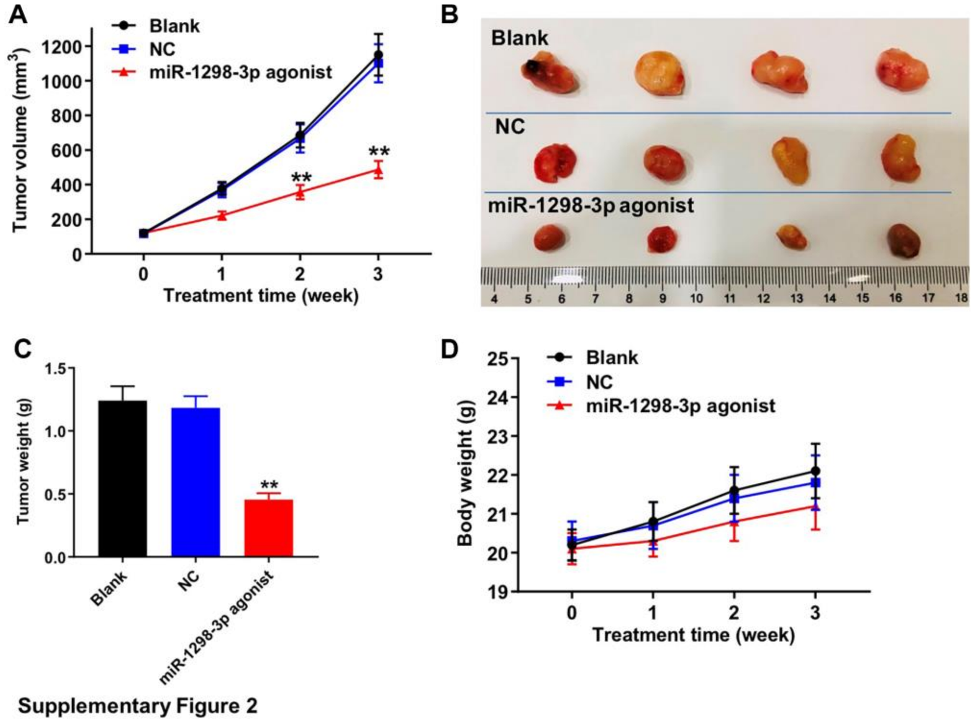

Supplementary Figure 2. Upregulation of miR-1298-3p inhibits tumorigenesis of U251 subcutaneous xenografts *in vivo*. U251 cells were subcutaneously injected into nude mice, and 50 nM miR-1298-3p agonist was directly injected into the tumors twice a week. (**A**) Xenograft tumor volume was monitored weekly. (**B**, **C**) Xenografts tumors were photographed and calculated. (**D**) The body weights of mice were monitored. **P < 0.01 compared with the NC group.
